# Is the thymidine labeling index a good prognostic marker in breast cancer?

**DOI:** 10.1186/1477-7819-5-93

**Published:** 2007-08-19

**Authors:** Ebru Sen-Oran, Vahit Ozmen, Ayhan Bilir, Neslihan Cabioglu, Mahmut Muslumanoglu, Abdullah Igci, Nese Guney, Mustafa Kecer

**Affiliations:** 1 Departments of General Surgery, Istanbul Medical School, Istanbul University, Istanbul, Turkey; 2 Department of Histology and Embryology, Istanbul Medical School, Istanbul University, Istanbul, Turkey; 3 Department of Oncology, Istanbul Medical School, Istanbul University, Istanbul, Turkey; 4 Department of Surgery, Memorial Hospital, Istanbul, Turkey

## Abstract

**Background:**

The aim of the present study was to determine the prognostic relevance of thymidine labeling index (TLI) in patients with breast cancer.

**Methods:**

TLI of the primary tumor was measured in 268 patients at the time of the surgical biopsy by an in vitro method.

**Results:**

Fifty-four patients had stage I disease, and 138 patients had stage II disease, and 76 patients had stage III disease. One hundred-four patients were found to have low TLI-index (<3%), and 164 patients had high TLI-index (≥3%). The median follow-up was 71.5 months (range, 6–138 months). The 5-year overall survival (OS) and disease free survival (DFS) rates was 84% and 74%, respectively. Lymph node involvement, tumor size more than 2 cm, high nuclear grade and estrogen receptor negativity were found to be associated with poorer DFS and OS rates. On subgroup analysis, however, the 5-year OS rate was significantly higher in the low TLI-group than in the high TLI-group in patients with stage I disease (100% vs 76%, p = 0.05).

**Conclusion:**

Our findings suggest that the prognostic significance of TLI appears to be limited to early breast cancer that might help to distinguish patients who need more aggressive adjuvant treatment.

## Background

The determination of prognosis of a patient with breast cancer is extremely important due to the complex biology of cancer. Great efforts have been made to separate patients who need agressive systemic treatment due to high-risk of recurrence, from those in whom loco-regional treatment is sufficient. For this purpose, increasing number of biological markers such as hormone receptors, bcl-2, p53 mutations, c-erbB2 over-expression, Ki-67, nuclear DNA ploidy, and microvessel density have been proposed as potential prognostic markers in breast cancer [1-5]. Many of these markers appeared to be promising in initial reports but eventually failed to maintain their predictive value on clinical outcome. Among these markers, the proliferative rate of tumor cells, as estimated by different approaches, has drawn great attention as a prognostic factor. Proliferative activity of the tumor cells utilizing H_3_-thymidine labeling index (TLI) has been a reliable and reproducible method. As a dynamic measurement of *de novo *DNA synthesis, TLI reflects the percentage of cells in the S-phase fraction of the cell cycle [6]. Although there are several studies that emphasize proliferative index of tumor could provide relevant information on prognosis of patient with breast cancer and on prediction of response to treatment, debate still remains [7-11]. The main reasons for the conflicting results might be due to the techniqual difficulties in quantifying TLI and the heterogenicity of patient series.

In this study, we investigated the prognostic value of TLI in our patient population with operable breast cancer by analyzing various associations between TLI and tumor characteristics and outcome by comparing with other previously established prognostic factors.

## Patients and methods

Between April 1993 and February 2000, 268 consecutive patients with operable breast cancer treated at the Breast Cancer Research and Treatment Unit at the Istanbul University, Istanbul Medical School, were retrospectively analyzed. The study was approved by the university ethics committee, and all participating patients gave informed consent. Patients with systemic metastases at the time of diagnosis (n = 33), and patients with neoadjuant chemotherapy (n = 35) were excluded from the study. Medical records were reviewed to collect the following data: age, menopausal status, type of surgery performed (mastectomy or breast conserving surgery), tumor characteristics (tumor size, nuclear and histological grade, histological type, presence of lymphovascular invasion, status of estrogen and progesterone receptors, presence of multifocality or multicentricity), nodal status, stage, adjuvant treatment (endocrine therapy, chemotherapy, radiotherapy), local and systemic recurrences, follow-up time. Histological and nuclear grades of the primary tumors were determined according to the Richardson-Bloom grading system [12]. The 6th edition of the AJCC Cancer Staging system was used for staging [13].

### Assessment of Thymidine Labeling Index

Thymidine labeling index was determined immediately after surgical biopsy of tumor samples obtained from patients with breast cancer as described before [9]. Briefly, the tumor was minced into 8–10 fragments of about 1 mm^3^. The minced fragments were placed in 2 mL of 199 medium (Biological Industries, Kibbutz Beit Haemek, Israel) containing 20% fetal calf serum (Biological Industries, Kibbutz Beit Haemek, Israel), streptomycin 100 microg/ml, penicillin 100 U/mL, and 6 micro Ci/mL H_3_-thymidine with specific activity 5 Ci/mol (Radiochemical Center, Amersham Life Science, UK). They were incubated for 1 hour at 37°C in shaker water bath. After the incubation period, the tumor fragments were washed 3 times in phosphate-buffered solution, and fixed in buffered 10% formalin solution dehydrated in alcohol, and embedded in parafin. Paraffin sections were obtained cut at 5 micron. Slides were coated with emulsion film (Ilford K2, Mobberley Cheshire, UK) in a dark room and exposed at 4°C for 3–5 days. Autoradiographies were then developed in D 19 b 5 minutes at 18°C, and fixed in a standart fixer. The slides were stained with hematoxylin and eosin at 4°C. A total of 1000–3000 cells were counted to determine the ratio of labeled cells. A tumor cell was considered labeled with thymidine when it contained at least 20 grains overlying the nucleus. Thymidine labeling index was estimated as the percentage of epithelial cells labeled with thymidine. Values less than 3% were considered as low TLI, whereas values equal to or more than 3% were considered high TLI based on previous studies [14-18].

### Follow-up

Patients were followed up with history and physical examination at least every 3 months for the first 2 years and then every 6 months for the next 2 years and then annually thereafter, if they were free of disease. Mammography of the breast along with chest X-ray, liver ultrasound, bone scintigraphy and biochemical screening were obtained in patients with high likelihood of recurrence once a year. Loco-regional and distant relapses were diagnosed by imaging techniques and/or biopsy.

### Statistical Analysis

The SPSS 10.1 software package (SPSS Inc., Chicago, IL) was used for statistical analyses. Patients were tabulated according to their TLI status whether they had tumors with low or high TLI. Associations between TLI and various factors such as patient and tumor characteristics and outcome were investigated. Chi-square test was used in univariate comparison analyses. Disease-free survival (DFS) time was considered as the interval between the date of first diagnosis of the tumor and the date of the first documented evidence of new disease manifestation in locoregional or distant sites. Overall survival (OS) time was defined as the interval between the first diagnosis of the tumor and the date of the last follow-up or death. Patients who were alive or had died of any cause were censored for analysis of OS. Kaplan-Meier survival test was used in survival analyses. Survival rates were compared by log-rank test. Variables that were found to be significant in univariate Kaplan-Meier survival analyses or thought to be clinically significant such as TLI were further evaluated in multivariate Cox regression model to determine the independent factors associated with OS or DFS rates. A p-value of less than or equal to 0.05 was considered to be statistically significant.

## Results

### Patient and tumor characteristics

Patient and tumor characteristics were shown in Table 1 and 2. The median age of patients was 50 years (range 23–87 years), and 161 patients (60.5%) were postmenopausal. One hundred eighty-two patients (67.9%) underwent modified radical mastectomy, and 86 patients (32.1%) had breast conserving surgery with complete axillary dissection. According to the AJCC staging criteria, 54 patients (20.1%) had stage I disease, 138 patients had stage II disease (51.5%), and 76 patients (28.4%) had stage III disease. Two-hundred five patients (76.5%) received adjuvant chemotherapy, and 187 patients with estrogen and/or progesterone receptor positivity (69.8%) received hormonal therapy. All patients with breast conservation and 114 patients with mastectomy had also radiation therapy followed by surgery.

**Table 1:** Associations of patient characteristics with the TLI status.

	**All patients**		**Patients with TLI-low**		**Patients with TLI-high**		**p-value**
	n	%	n	%	n	%	

**Age**							0.900
≤50 years	136	50.7	52	38.2	84	61.8	
>50 years	132	49.3	52	39.4	80	60.6	
**Menopausal status**							0.559
Premenopausal	105	39.5	38	36.2	67	63.8	
Postmenopausal	161	60.5	64	39.8	97	60.2	
**Tumor**							0.306
pT1	103	38.4	44	42.7	59	57.3	
pT2+pT3+pT4	165	61.6	60	36.4	105	63.6	
**Node**							0.448
pN0	114	42.5	41	36.0	73	64.0	
pN(+)	154	57.5	63	40.9	91	59.1	
**Stage**							0.596
I	54	20.1	18	33.3	36	66.7	
II	138	51.5	54	39.1	84	60.9	
III	76	28.4	32	42.1	44	57.9	
**Type of surgery**							0.330
Breast conservation	86	32.1	37	43.0	49	57.0	
Mastectomy	182	67.9	67	36.8	115	63.2	
**Radiation therapy**							0.911
Yes	200	74.6	78	39.0	122	61.0	
No	68	25.4	26	38.2	42	61.8	
**Hormonal Therapy**							0.021
Yes	187	69.8	81	43.3	106	56.7	
No	81	30.2	23	28.4	58	71.6	
**Chemotherapy**							0.451
Yes	205	76.5	77	37.6	128	62.4	
No	63	23.5	27	42.9	36	57.1	

**Table 2:** Associations of tumor characteristics with the TLI status.

	**All patients**		**Patients with TLI-low**		**Patients with TLI-high**		**p-value**
	n	%	n	%	n	%	

**Histologic type**							0.556
Invasive ductal	182	67.9	72	39.6	110	60.4	
Invasive lobular	18	6.7	9	50.0	9	50.0	
Mixed ductal&lobular	50	18.7	18	36.0	32	64.0	
Other	18	6.7	5	27.8	13	72.2	
**Multifocality + Multicentricity**							0.456
Yes	34	12.7	11	32.4	23	67.6	
No	234	87.3	93	39.7	141	60.3	
**Nuclear Grade**							0.008
1+2	177	66	79	44.6	98	55.4	
3	91	34	25	27.5	66	72.5	
**Histologic Grade**							0.999
1+2	136	50.7	53	39.0	83	61.0	
3	132	49.3	51	38.6	81	61.4	
**Lymphovascular invasion**							0.753
Yes	206	76.9	81	39.3	125	60.7	
No	62	23.1	23	37.1	39	62.9	
**Estrogen receptor (ER) status**							0.124
ER-positive	173	64.6	73	42.2	100	57.8	
ER-negative	95	35.4	31	32.6	64	67.4	
**Progesteron receptor (PR) status**							0.134
PR-positive	69	25.7	32	46.4	37	53.6	
PR-negative	199	74.3	72	36.2	127	63.8	

One hundred-four patients (38.8%) were found to have low TLI, and 164 patients (61.2%) had high TLI. When associations between TLI and other patient or tumor characteristics were investigated, patients with high TLI were less likely to receive hormonal therapy than patients with low TLI (low TLI-group, 56.7%, vs. high TLI-group, 43.3%, p < 0.021). Furthermore, patients with high nuclear grade were also more likely to have high TLI values compared with patients with low or intermediate nuclear grade (low & intermediate NG, 55.4%, vs. high NG, 72.5%, p = 0.008). However, no other significant associations could be found between TLI and other parameters (Table 1 and 2).

### Outcome

The median follow-up was 71.5 months (range, 6–138 months). The 5-year overall survival (OS) and disease free survival (DFS) rates were 84% and 74%, respectively. During the follow-up period, loco-regional recurrence was observed in 8 of 104 (7.7%) patients with low TLI and in 7 of 164 patients (4.3%) with high TLI. Moreover, distant metastases were found in 26 patients (25.0%) among patients with low TLI, and in 26 patients (28.7%) among patients with high TLI, respectively.

**Figure 1:**
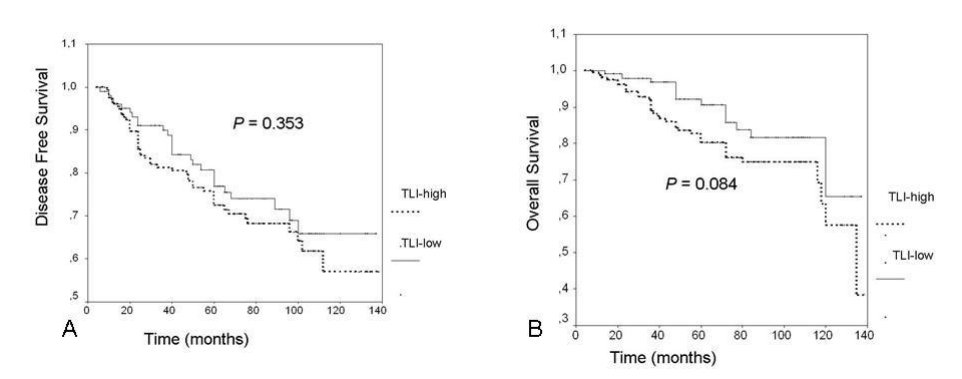
**A) **No significant difference could be found in 5-year disease free survival rates between all patients with low TLI and high TLI (p = 0.353). B) Patients with low TLI had higher 5-year OS rates than patients with high TLI but it did not reach statistical significance (p = 0.084).

**Figure 2:**
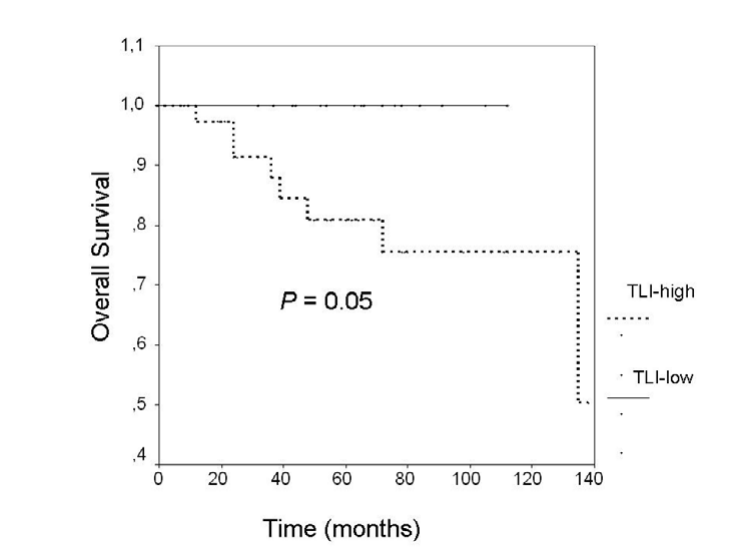
Among patients with stage I disease, patients with low TLI were found to have improved 5-year overall survival rates compared with patients with high TLI (p = 0.05).

**Figure 3:**
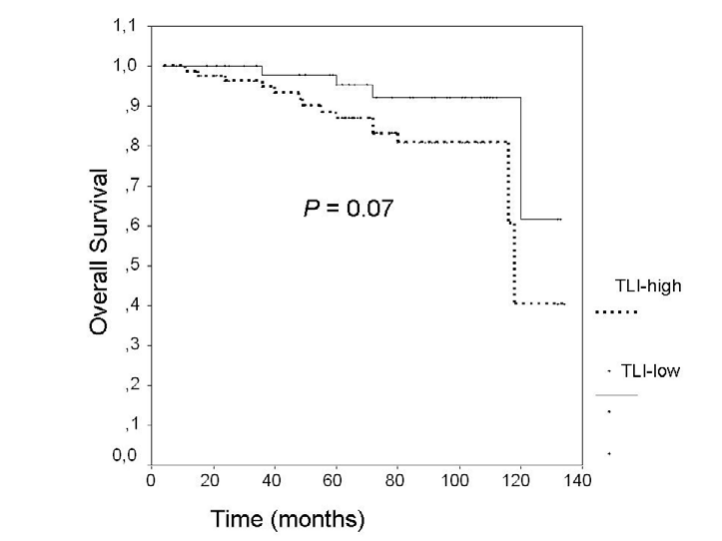
Among patients with stage II disease, patients with low TLI were found to have improved 5-year overall survival rates compared with patients with high TLI, but it did not reach the statistical significance (p = 0.07).

As would be expected from previous numerous studies, lymph node involvement, tumor size more than 2 cm, high nuclear grade and estrogen receptor negativity were found poor prognostic factors associated with decreased DFS and OS rates compared with others (Table 3). Presence of lymphovascular invasion (LVI) in breast tumors was associated with decreased 5-year-DFS and OS rates in patients compared with others, but these associations did not reach the statistical significance (LVI+, 69.2% vs LVI-, 74.7%, p = 0.088 for 5-year DFS, and LVI+, 63.5% vs LVI-, 70.6%, p = 0.058 for 5-year OS). Furthermore, no significant difference could be found in 5-year disease free survival rates between all patients with low TLI and high TLI (76.9% for low TLI vs 72.4% for high TLI, p = 0.353, Figure 1a). On the other hand, patients with low TLI were found to have higher 5-year OS rates than patients with high TLI but it did not reach statistical significance (75.4% for low TLI vs 64.8% for high TLI, p = 0.084, Figure 1b). On subgroup analyses among patients with stage I disease however, patients with low TLI were significantly found to have improved 5-year OS-rates compared with patients with high TLI (low TLI, 100% vs high TLI, 76%, p = 0.05, Figure 2). Among patients with stage II similarly, patients with low TLI were found to have better 5-year OS rates compared with patients with high TLI, but this association did not reach the statistical significance (95.4% for low TLI vs 89.7% for high TLI, p = 0.07, Figure 3). No other significant associations could be found between TLI and 5-year OS or DFS rates in other subgroups as shown in Table 4.

**Table 3:** Associations of patient and tumor characteristics with the disease free survival (DFS) and overall survival (OS) rates.

Variable	5-year DFS rate (%)	P-value	5-year OS rate	P-value
**Age**		0.745		0.319
≤50 years	74.3		76.1	
>50 years	74.1		61.9	
**Tumor**		0.002		0.001
pT1	83.4		81.9	
Other (pT2+ pT3+pT4)	69.0		61.7	
**Node**		0.0001		0.004
pN0	85.1		79.5	
pN1+pN2+pN3	66.0		61.4	
**Nuclear Grade**		0.007		0.032
1+2	76.4		70.5	
3	67.7		66.7	
**Histologic Grade**		0.080		0.093
1+2	78.9		75.9	
3	67.2		61.2	
**Estrogen receptor status**		0.030		0.016
ER-positive	78.1		74.3	
ER-negative	64.6		59.1	
**Progesterone receptor status**		0.331		0.567
PR-positive	79.0		71.1	
PR-negative	71.4		68.2	
**Lymphovascular invasion**		0.088		0.058
Yes	69.2		63.5	
No	74.7		70.6	
**TLI status**		0.353		0.084
Low TLI	76.9		75.4	
High TLI	72.4		64.8	

**Table 4:** Associations between TLI status and OS or DFS rates of the patient subgroups

	5-year DFS rate (%)	P-value	5-year OS rate (%)	P-value
Patients with adjuvant chemotherapy (n = 205)		0.272		0.224
Low TLI	75		89	
High TLI	68		80	
				
Patients without adjuvant chemotherapy (n = 63)		0.663		0.634
Low TLI	83		95	
High TLI	89		96	
				
Patients with adjuvant hormonal therapy(n = 187)		0.512		0.824
Low TLI	79		89	
High TLI	78		89	
				
Patients without adjuvant hormonal therapy (n = 81)		0.933		0.164
Low TLI	69.4		88.9	
High TLI	63.5		74.4	
				
Patients with stage I (n = 54)		0.474		0.05
Low TLI	88.2		100	
High TLI	78.7		76.4	
				
Patients with stage II (n = 138)		0.464		0.070
Low TLI	79.8		95.4	
High TLI	74.2		89.7	
				
Patients with stage III (n = 76)		0.526		0.800
Low TLI	65.7		76.3	
High TLI	63.2		73.4	
				
Patients with node-negative disease (n = 114)		0.994		0.188
Low TLI	83.5		92.6	
High TLI	86.1		88.6	
				
Patients with node-positive disease (n = 154)		0.142		0.113
Low TLI	72.6		86.4	
High TLI	61.2		73.6	

When variables that were found to be significant in univariate Kaplan-Meier survival analyses or thought to be clinically significant were further evaluated in multivariate Cox regression model, node positivity (HR [vs. other] = 2.6; 95% CI, 1.5–4.5; *P *= 0.001), and tumor size > 2 cm (HR [vs. other] = 2.1; 95% CI, 1.2–3.7; *P *= 0.010) were independent predictors of decreased 5-year DFS (Table 5). Similarly, node positivity (HR [vs. other] = 2.3; 95% CI, 1.2–4.4; *P *= 0.012), and estrogen receptor negativity (HR [vs. other] = 1.9; 95% CI, 1.0–3.4; *P *= 0.036) were also found as independent predictors of decreased 5-year OS (Table 6). However, the other factors including TLI failed to show a significant independent predictive value for DFS or OS on multivariate analyses in this study group (Table 5 and 6).

**Table 5:** Multivariate cox regression analyses for factors affecting disease free survival rates.

Variable	Hazard ratio	95% CI	*P *value
Node positivity (positive vs negative)	2.6	1.5–4.5	0.001
Tumor size >2 cm (vs ≤2 cm)	2.1	1.2–3.7	0.010
Nuclear grade (high vs low&intermediate)	1.1	0.6–1.8	0.846
Histologic grade (high vs low&intermediate)	1.6	1.0–2.7	0.060
Lymphovascular invasion (positive vs negative)	1.3	0.8–2.2	0.309
Estrogen receptor (negative vs positive)	1.4	0.9–2.3	0.140
TLI (high vs low)	1.2	0.7–1.9	0.522

**Table 6:** Multivariate cox regression analyses for factors affecting overall survival rates.

Variable	Hazard ratio	95% CI	*P *value
			
Node positivity (positive vs negative)	2.3	1.2–4.4	0.012
Tumor size >2 cm (vs ≤2 cm)	1.9	1.0–3.9	0.058
Nuclear grade (high vs low&intermediate)	1.2	0.6–2.3	0.591
Histologic grade (high vs low&intermediate)	1.6	0.8–3.0	0.160
Lymphovascular invasion (positive vs negative)	1.6	0.8–3.0	0.160
Estrogen receptor (negative vs positive)	1.9	1.0–3.4	0.036
TLI (high vs low)	1.6	0.8–3.0	0.161

## Discussion

Several prognostic factors such as tumor size, lymph node involvement, nuclear and histologic grade, and hormone receptor status are commonly used together in predicting the clinical outcome of patients with breast cancer rather than a single parameter. Investigations are going on to find out an ideal prognostic factor which separates the patients into low risk and high risk groups in terms of the probability of recurrence. In the meta-analysis by Mirza *et al*. [19], studies with sample size more than 200 and follow-up more than 5 years were evaluated and tumor size, tumor grade, cathepsin-D, Ki-67, S-phase fraction, mitotic index, and vascular invasion were found to be associated with survival outcome in patients with early-stage node-negative breast cancer. Because of the techniqual difficulties and variations in the measurement of many of these factors, tumor size and tumor grade have been accepted as the only markers that currently had widespread clinical usefulness in these patient group [19].

The prognostic and predictive relevance of tumor cell proliferation expressed as TLI has been reported in many studies [7-10]. Highly proliferative tumors are generally related to shorter disease free and overall survival rates. Significant correlation between the TLI value and the aggressiveness of breast carcinoma was observed in the majority of the studies, and TLI was found to be the most significant prognostic indicator in regards to the survival [4,10,20]. According to the Meyer's study [20] including 227 operable breast cancer who were treated by radical mastectomy, the low TLI-group had a probability of relapse risk of 20% at 4 years, in contrast to 52% for the high TLI-group. Similarly, Tubiana *et al*. [7] reported the recurrence rates of breast carcinoma as 25% and 62% in patients with low and high TLI groups, respectively at a 10-year follow-up.

We previously reported that TLI was a strong independent prognostic factor affecting OS in locally advanced breast cancer among the other established clinical and biological parameters [9]. In this study, we investigated the prognostic relevance of TLI in 268 operable breast cancer. We could not demonstrate any significance of TLI to predict survival in univariate and multivariate analyses in this study group in concordance with our previous study including 155 patients [21]. Furthermore, we were unable to show any association between TLI and survival in 114 patients with lymph node negative disease in the current study. However, on subgroup analyses according to the stage, we interestingly found that low TLI may be a predictor of improved overall survival in breast cancer with stage I disease in concordance with previous studies [10]. This disconcordance might be due to the different tumor sizes along with other clinicopathologic features of patients with lymph node negative disease compared to patients with stage I disease in the current study. Among patients with stage II disease moreover, better overall survival rates were determined in patients with low TLI compared with patients with high TLI, but this association did not reach the statistical significance. Therefore, our results suggest that TLI may be a useful prognostic marker in early-stage breast carcinoma to determine further therapeutical interventions after surgery.

Besides its prognostic relavance, predictive value of TLI on clinical response to different therapautic agents is also the subject of debate [9,22-24]. Some retrospective studies have shown no relation between TLI and response to neoadjuvant chemotherapy in patients with locally advanced breast cancer [9]. A recent randomized prospective study published by Amadori *et al*. [23] have reported that a DFS advantage of adjuvant CMF exists in node negative patients with high TLI values. On the current study, patients were separately analysed in subgroups according to the postoperative therapeutic approach in an attempt to eliminate the discrepancy between groups, and no significant associations could be found between TLI and survival on these subgroup analyses. Further studies are required to determine the predictive value of TLI on clinical response to treatment.

In the present study, high TLI value was detected more frequently in breast cancer with high nuclear grade that might be an indicator of a more aggressive tumor. Both rapid proliferative capacity and increased nuclear grade indicate the agressiveness of breast carcinoma, as reported in many other studies previously [6,20,21]. However, we could not find any other significant associations between TLI and tumor or patient characteristics such as tumor size or lymph node status. Some investigators have found positive association between TLI and tumor size as opposed to our study [6]. Furthermore, consistent with our data, other studies reporting analyses of large series have also failed to show an association between the TLI and lymph node status [17,20,25].

Potential causes for conflicting results in the studies on TLI might be attributable to the difficulties in the methodological procedure to determine H_3_-TLI, to the lack of a standard cut-off point of TLI to classify patients as low-TLI and high-TLI group, and to the heterogenicity of patient series including differing prognostic factors, treatment modalities, and follow-up intervals. In this study, we accepted the value of 3% for TLI as the cut-off point in concordance with previous studies that found TLI as a significant prognostic factor on the large series of primary breast cancer by using this cut-off value [14-18]. Due to the some difficulties in the methodology such as the necessity to perform the assay on fresh samples and the absence of availability in peripheral institutions, this procedure has not been used widely. Therefore, S-phase fraction has been used more commonly to measure the proliferative activity of breast tumors although there are controversial reports of its prognostic value on survival of breast cancer patients [19,26,27]. In order to facilitate the assay of proliferative index, some techniqual modifications were performed in our clinic, and we currently use the thymidine analog bromodeoxyuridine (BrdUrd) instead of H_3_-TLI since 2000.

Our results suggest that the prognostic significance of TLI appears to be limited in breast cancer except early breast cancer to distinguish patients who need more aggressive adjuvant treatment. However, other prognostic factors rather than proliferative index should be generally considered in planning of the systemic treatment of the patients. Investigations are still going on to find the ideal prognostic factors in breast carcinoma that would assist clinicians in decision-making process to select the appropriate therapeutic interventions. For this purpose, microarray-based gene expression profiling of human breast cancer recently emerged as novel screening techniques to estimate patient's risk of recurrence Using a multistep approach, a 21-gene assay (Oncotype DX) was recently developed for use in paraffin-embedded tumor tissue to predict risk for distant recurrence or death in lymph node-negative breast cancer patients [28]. Approximately 250 genes, selected from the published literature, genomic data-bases, pathway analysis, and from microarray-based gene expression profiling experiments, were considered as candidates. The final gene list (16 cancer-related and five reference genes) and summary score (Recurrence Score) algorithm for this assay were developed by analyzing the results of three independent preliminary breast cancer studies conducted in a total of 447 patients [29]. All these microarray-based gene expression analyses of breast cancer include also proliferation related-gene analyses. By using this assay, a recent study demonstrated that the Recurrence Score was strongly associated with risk of breast cancer death among ER-positive, and lymph node-negative patients not treated with chemotherapy [30].

## Conclusion

Along with the novel microarray-based gene expression analyses, TLI may be useful as a prognostic indicator of the biological agressiveness of tumor in patients of early-stage, especially those with stage I disease to select patients who could benefit from systemic therapies including chemotherapy. Further prospective, large-scale studies are needed to reach a general consensus on the relevance of H_3_-TLI as a prognostic or predictive indicator in breast cancer.

## Competing interests

The author(s) declare that they have no competing interests.

## Authors' contributions

ESO drafted the manuscript.

VO conceived of the study, and participated in its design and coordination and helped to draft the manuscript.

NC participated in the design of the study and performed the statistical analyses and helped to draft the manuscript.

AB performed the thymidine labeling index assays of the breast tumors that were included into the study.

NG helped to collect the data (outcome etc) of the patients.

MM, AI, and MK, provided the breast tumors that were included into the study, and they all critically reviewed the manuscript.

All authors read and approved the manuscripts.
